# Addictive Behaviors During the 2022 FIFA World Cup: A Qualitative Study of Patients and Healthcare Staff at a Substance Use Disorder Facility

**DOI:** 10.3390/ijerph23050586

**Published:** 2026-04-30

**Authors:** Khalifa Al Kuwari, Izzeldin Ibrahim, Abdulaziz Farooq, James England, Perla ElMoujabber, Rama Kamal, Karim Chamari, Vidya Mohamed-Ali, Mohammad Al-Maadheed

**Affiliations:** 1 Naufar Center, Doha P.O. Box 93097, Qatar; khalifa.alkuwari@naufar.com (K.A.K.); james.england@naufar.com (J.E.); perla.elmoujabber@naufar.com (P.E.); rama.kamal@naufar.com (R.K.); karim.chamari@naufar.com (K.C.); 2 Aspetar Orthopedic and Sport Medicine Hospital, Doha P.O. Box 29222, Qatar; mohammed.farooq@aspetar.com; 3 Centre of Metabolism & Inflammation, Division of Medicine, University College London, London WC1E 6BT, UK; v.mohamed-ali@ucl.ac.uk (V.M.-A.); m.maadheed@ucl.ac.uk (M.A.-M.)

**Keywords:** addictive behavior, mass-gathering events, Naufar, Qatar

## Abstract

**Highlights:**

**Public health relevance—How does this work relate to a public health issue?**

- Our study investigates substance use disorders (SUDs) as a significant public health concern by evaluating the influence of the 2022 FIFA World Cup on substance use patterns, relapse risk, and engagement with addiction treatment in Qatar.
- The findings demonstrate that large-scale mass gatherings can alter social and environmental conditions, thereby affecting vulnerable populations and reinforcing the context-sensitive nature of SUDs as a public health issue.

**Public health significance—Why is this work of significance to public health?**

- Our study addresses a gap in global evidence regarding the impact of mass gatherings on addiction-related health outcomes, with direct implications for prevention and harm reduction strategies.
- It provides real-world insights into the adaptation of specialized addiction services during a mega-event, offering lessons that are applicable to public health systems in future host countries.

**Public health implications—What are the key implications or messages for practitioners, policy makers and/or researchers in public health?**

- Our study highlights the necessity for proactive and flexible addiction treatment planning during mass gatherings, including relapse prevention and continuity-of-care measures for individuals at high risk.
- The findings support the integration of substance use and mental health considerations into mass-gathering preparedness frameworks and underscore the need for further research on long-term system- and patient-level impacts.

**Abstract:**

Background: Mega-events like the FIFA World Cup (FWC) present unique and substantial challenges for individuals in recovery from substance use disorders (SUDs), primarily by increasing the risk of relapse. We employed a qualitative design using reflexive thematic analysis to explore the behavior of patients with SUDs during the 2022 FWC and to evaluate institutional strategies for mitigating related risks. Methods: We purposively sampled 32 participants who were present at the Naufar Center during the 2022 FWC: (i) thirteen adult patients with SUDs who were receiving treatment, and (ii) nineteen healthcare practitioners. Semi-structured patient interviews were conducted, and focus group discussions were held with a multidisciplinary team, including psychologists, nurses, and physicians. Individuals’ experiences regarding patterns in substance use behavior, environmental triggers, and the effects of institutional interventions were examined. Thematic analysis was employed to identify patterns, risks, and effective strategies. Results: Most patients maintained abstinence and only had cravings for alcohol. Triggers included public celebrations, emotional excitement, and the increased availability of addictive substances. Psychologists and physicians reported signs of behavioral destabilization; nurses observed some behavioral changes and noted logistical challenges. The participants acknowledged the supportive measures provided by Naufar, including the accessibility of clinical services, individualized therapy, social and recreational programming, and protective fan zones, which enabled them to participate in various activities during the event. Conclusions: The 2022 FWC created considerable psychological and environmental triggers for high exposure to alcohol and other substances. The supportive structured activities and tailored interventions were helpful in mitigating the risk of relapse, maintaining treatment engagement and ensuring recovery. Further research is required to explore the implications for recovery-oriented practices during culturally and socially high-risk events.

## 1. Introduction

Substance use disorders (SUDs) represent a critical public health threat, imposing a considerable burden on society and increasing morbidity and mortality [1]. Addiction is not a single issue but a complex condition influenced by numerous factors, including genetics and environmental influences [2]. Emotional stimulation, social exposure, and/or substance availability can increase the prevalence of SUDs and the risk of relapse. Mass-gathering events (MGEs), despite their social and cultural importance, have a significant impact on addictive behavior and the risk of relapse for individuals with SUDs [3,4,5,6]. The FIFA World Cup (FWC) is a major sporting event that attracts millions of people from around the world to a host venue and is characterized by the increased availability and use of alcohol and other illicit substances [6,7]. Studies have described a link between MGEs, like the FWC, and a rise in substance use among vulnerable populations. Patients recovering from or receiving treatment for SUDs are more likely to relapse during MGEs than during other times of the year [8,9,10,11]. The addictive behavior patterns of patients with SUDs change according to the socio-cultural circumstances of each community [12]. Studies on FWCs have highlighted how these events provide opportunities for community participation and cultural promotion while gathering people from diverse backgrounds; this increases exposure to high-risk environments and relapse triggers for patients with SUDs [10,12].

International sporting events provide excellent opportunities for host nations to foster development [13]. Countries in the Arabian Peninsula are actively hosting major sporting events as a strategic tool for both political and socio-economic development [14,15,16,17,18]. The 2022 FWC hosted in Qatar brought about extraordinary social gatherings, public celebrations, and increased access to illicit substances such as alcohol. For addiction treatment centers, the challenge is in balancing patient engagement with relapse prevention, particularly in the face of heightened emotional stimulation and external environmental triggers.

The Naufar Center, as a leading specialized center for addiction treatment and rehabilitation in Qatar [19,20,21], recognized the risks posed by the 2022 FWC and proactively implemented a series of structured evidence-based interventions to maintain recovery momentum among its patients [22]. The primary aim of our exploratory study was to identify the impact of the 2022 FWC on patients’ addiction behaviors at Naufar and evaluate the institution’s response strategies. The study reports findings from in-depth interviews with patients and three staff-based focus groups, exploring both observed patient behavior (Theme 1) and the institutional strategies used to manage cravings and maintain healing engagement (Theme 2).

## 2. Methods

### 2.1. Study Design

Our study was conducted at the Naufar Center, focusing on adult patients undergoing treatment for SUDs during the 2022 FWC. We employed a qualitative design with reflexive thematic analysis [23,24]. to investigate patients’ addictive behaviors, motivational drivers, and coping mechanisms during this period with an aim of capturing the lived experiences of patients receiving SUD treatment and gathering professional insights from the clinical staff involved in their care.

### 2.2. Research Team Description

Our research team consisted of a multidisciplinary team of therapists and staff members, including clinical psychologists, physicians, nurses, and academic researchers with expertise in addiction rehabilitation and qualitative inquiry. Patient interviews were conducted by an experienced clinical psychologist trained in qualitative interviewing techniques, while trained clinical staff members facilitated focus group discussions. The team collaborated closely throughout data collection and analysis, ensuring consistency, rigor, and reflexivity in the interpretation of findings.

### 2.3. Recruitment of Participants

Participants were recruited using a purposive sampling technique based on predefined inclusion criteria, focusing on adult patients receiving treatment for SUDs at Naufar Center during the 2022 FWC. Recruitment continued until data saturation was achieved, defined as the point at which no new concepts emerged after conducting two additional interviews [25,26]. Clinical staff, including psychologists, nurses, and physicians, were invited to participate in focus groups due to their direct involvement in patient care during the tournament period.

### 2.4. Data Collection Methods

Two complementary data collection strategies were used. The first involved in-depth semi-structured interviews with thirteen patients. These interviews elicited a comprehensive narrative describing participants’ substance use behaviors, coping mechanisms, and institutional support during the 2022 FWC. Interviews were audio-recorded, and participants gave their consent in a private setting to ensure confidentiality. The second strategy involved organizing three focus group discussions with five to eight members of the multidisciplinary clinical staff (including nurses, psychologists, and physicians). These sessions sought to understand professional perspectives regarding both patient behaviors and the institutional actions taken during the event. Trained staff moderated the focus groups. All sessions were audio-recorded and transcribed before data analysis. We extracted patients’ demographic and clinical information directly from their electronic medical records. All necessary ethical approvals were secured prior to data collection.

### 2.5. Interview Schedule Development and Content

We developed the interview schedule based on a review of existing literature and clinical expertise. Focus group scenarios consisted of open-ended questions designed to examine substance use behaviors during the 2022 FWC, motivational factors for using or abstaining from illicit substances, coping strategies, and perceptions of Naufar’s institutional interventions. The schedule was piloted through small focus group sessions with selected participants to refine the wording and ensure it was culturally and contextually relevant. The flexible interview structure encouraged participants to elaborate on emergent themes, which was crucial for ensuring that their perspectives were accurately represented.

### 2.6. Strategies to Eliminate Bias

To eliminate the possibility of potential bias, we implemented measures to enhance the accuracy and credibility of the study, which included engaging in reflexive journaling to acknowledge and mitigate personal biases. We achieved data triangulation by incorporating diverse perspectives from both patients and clinical staff. The co-investigators reviewed and refined the coding frameworks to ensure consistency in data collection and analysis. The recruitment process was guided by the principle of data saturation to ensure comprehensive thematic coverage. A strict ethical methodology was implemented, ensuring all participants provided informed consent and establishing secure data management protocols. These steps were crucial for ensuring confidentiality and eliminating risks of coercion or breaches of anonymity.

### 2.7. Data Analysis

In our study, the data were coded using a combination of grounded theory, thematic analysis, and phenomenological analysis. Grounded theory facilitated developing new insights rather than testing existing ones, while phenomenological analysis enabled the exploration of patients’ lived experiences and perceptions of behavioral changes. Thematic analysis, on the other hand, identified recurrent themes and patterns across the dataset. All data were analyzed and coded using MAXQDA software (VERBI GmbH, Berlin, Germany, version 24.6.0, 2024) [27,28] in three stages. In the first stage, initial codes were generated based on the interview guide. In the second stage, overlapping themes were merged, and broader categories were divided into meaningful subcodes. In the third stage, the coding framework was re-examined using a randomly selected transcript and reviewed by the co-investigators before being applied to the full dataset. An inductive approach was maintained throughout, allowing new codes to be added when novel ideas emerged. Themes were refined in discussion with the research team, and illustrative quotes were used to highlight key findings. Frequency counts and code comparison analyses were also conducted to identify commonly occurring themes and relationships among participants’ responses. Thematic content analysis, as outlined by Braun and Clarke, was employed to systematically identify and interpret patterns across the datasets. Transcripts were analyzed using MAXQDA version 24.6.0.

### 2.8. Ethical Considerations

The study commenced only after obtaining the required ethical approval for research involving human participants (Ministry of Public Health registered IRB: E202401074). All participants provided informed consent. Confidentiality and anonymity were strictly maintained throughout the data collection process. Furthermore, secure data management practices were implemented to protect sensitive information.

## 3. Results

Thirteen male patients receiving SUD treatment at the Naufar Center (mean age: 35.2 years (SD: ±8.7)) were recruited. The most common drugs used by the participants were opioids (69.2%), alcohol (15.4%), and methamphetamines (Shaboo) (15.4%). Detailed information about the participants is presented in Table 1. Nineteen healthcare practitioners participated in the focus group discussions, as shown in Table 2. Based on the research objectives, the results were organized around two core thematic pillars related to the interventions: Theme 1: “The impact of the 2022 FWC on patients’ addictive behavior”. Theme 2: “Naufar’s institutional strategies during the 2022 FWC”.

### 3.1. Theme 1: The Impact of the 2022 FWC on Patients’ Addictive Behavior

The 2022 FWC served as a double-edged catalyst for patients in recovery. While the structured environment of the Naufar Center and the communal spirit of the event acted as protective “social scaffolding”, the environmental overstimulation and availability of substances (particularly alcohol) presented significant psychological hurdles for patients.

#### 3.1.1. Behavioral Patterns: Abstinence vs. Environmental Triggers

Most patients (9/13) maintained sustained abstinence throughout the tournament. For some patients, the FWC period was a milestone of *“normalcy,”* with participants (P2, P3, P13) describing it as their first experience of a fulfilling life without substance use.

Clinical insights from focus groups revealed greater complexity in these narratives. Many inpatients reported coping well during the FWC. However, physicians and psychologists observed heightened emotional volatility and agitation, frequently linked to patients’ anticipation of public festivities. Clinicians identified alcohol as the primary substance associated with cravings, particularly among those in sustained recovery who tended to minimize external triggers. Nurses observed that indirect cravings might manifest as repeated requests for home medication, missed appointments, or other disruptions to routine, rather than as an explicit urge to use substances: *“The low attendance in the daily schedule, especially in the morning”*. Psychologists noted that participation in fan zones inside Naufar was seen as positive, and patients were committed and disciplined within the controlled setting: *“At any time, the patient can see a therapist immediately”.*

Simultaneously, cravings during the FWC were often described as manageable, with 7 of 13 patients attributing this to the therapeutic environment and strong group cohesion. Peer support was particularly valued. Participants P6 and P12 described a *“protective pack”* approach, deliberately remaining in groups as they passed through crowded celebration areas, such as Souq Waqif and Lusail, to mitigate risk and enhance safety. Clinical support was also considered vital. Participants reported that pharmacological treatments, including Suboxone® (2, 4, 8 and 12 mg—Indivior Inc., Chesterfield Country, VA, USA), and structured cognitive methods effectively redirected and contained urges to use. As P11 stated, these interventions helped to *“keep the idea away”.* P8, P9 and P12 stated, *“the atmosphere in Naufar was nice”.* P2 stated, *“when I come to Naufar, all the negative energy drains from me”*.

#### 3.1.2. Psychological Resilience and Vulnerabilities

Our results reveal a stark contrast between those who felt protected by the “Naufar bubble” and those who felt the weight of external pressures (Table 3).

#### 3.1.3. Lessons in Long-Term Recovery

The 2022 FWC period provided critical “lived lessons” for the participants. The most prominent takeaway was the danger of isolation and boredom, with P10 explicitly stating, *“Free time is what kills a person.”* Patients concluded that recovery in a high-stimulus environment requires:

1. Selective Socialization: Avoiding “unsafe” environments and old peer groups (P6).
2. Integration: Using sports as a tool for societal reintegration (P13).
3. Humility: Accepting the guidance of advisors rather than relying solely on willpower (P2).

Summary of key findings: The 2022 FWC acted as a stress test for recovery in our participants. Success was not merely the absence of substance use but the presence of active engagement. Relapse (as seen with P5) was closely tied to environmental availability and emotional deterioration, whereas success was tied to *“group-guarded”* exposure to the festivities.

### 3.2. Theme 2: Naufar’s Institutional Strategies During the 2022 FWC

Naufar’s approach during the tournament was characterized by a shift from traditional clinical boundaries to a “total environment” strategy. By blending high-stakes entertainment with therapeutic control, the institution successfully neutralized environmental triggers while fostering a sense of social inclusion (Table 4).

#### 3.2.1. The “Fan Zone” as a Therapeutic Buffer

The cornerstone of Naufar’s strategy was the creation of internal fan zones. This innovation allowed patients to participate in the national excitement of the World Cup without the high-risk exposure to alcohol or triggering social circles found in public venues. From a patient experience perspective, participants (P8, P12) felt a sense of belonging, noting that the center provided a *“beautiful atmosphere”* where they could watch games regardless of ticket status. Psychologists and physicians utilized these zones as controlled exposure tools, providing *“positive distractions”* and keeping patients engaged from *“10 a.m. to 10 p.m.”* to mitigate the risks of boredom and isolation.

#### 3.2.2. Adaptive Clinical and Psychosocial Services

The institution transitioned to a more fluid, accessible service model to meet the unique stressors of a mega-event.

#### 3.2.3. Strategic Human Resource Deployment

A defining feature of the period was the high-visibility presence of staff. Some patients (e.g., P7) specifically noted off-duty staff joining activities voluntarily, which humanized the recovery process and strengthened the therapeutic alliance. Moreover, nurses and psychologists maintained a constant presence to manage medication timing and immediate craving-related interventions, ensuring that the *“joyful gatherings”* did not compromise clinical stability. However, a minor gap was raised by a patient (P5) who noted reduced medical staffing during some shifts, perceiving a lean toward *“entertainment over treatment”*; this suggests the need to maintain a delicate balance between a festive atmosphere and clinical rigor when considering such experiences surrounding major global events for patients with SUDs.

#### 3.2.4. Tailored Relapse Prevention and Innovation

Naufar’s strategies extended beyond the center’s walls through proactive outreach and lifestyle coaching. From an environmental modification perspective, clinicians encouraged patients to change phone numbers and avoid *“old friends,”* a strategy adopted by P6 to maintain a clean break from active users. Staff also helped patients reframe FWC temptations not as *“missed pleasure”* but as *“traps”*, focusing instead on the superior joy of a sober life (P7). Furthermore, physicians recommended flexible work hours for employed patients, recognizing that occupational stress during the tournament could serve as a major relapse trigger.

Summary of key findings: Naufar Center was successfully transformed from a traditional clinic into a protective social ecosystem. By “bringing the World Cup inside”, the institution eliminated the “fear of missing out” that often drives relapse during major cultural events, replacing it with a structured, staff-supported “sober celebration.”

The success of Naufar’s intervention during the 2022 FWC was rooted in the alignment between clinical intent and patient perception. The table below (Table 5) illustrates how specific strategies translate into recovery milestones.

## 4. Discussion

Our study provided important insights into how large-scale cultural events, such as the 2022 FWC, can influence addictive behaviors among patients undergoing treatment for SUDs. Overall, the tournament period was associated with sustained abstinence and reduced cravings among most participants. Structured engagement at Naufar Center, combined with strong peer and social support, created a protective environment that enabled patients to resist relapse and experience sobriety as a meaningful lifestyle change. These findings align with existing evidence emphasizing the importance of structured therapeutic settings and social connectedness as protective factors in recovery [29].

Despite these positive outcomes, our results also highlighted the heterogeneity of patient responses. A subset of participants reported persistent cravings, and one individual relapsed during the tournament period, citing environmental triggers and emotional distress. This underscores the vulnerability of some patients even within protective environments, reflecting the complex and individualized nature of addiction recovery [2]. Psychological distress, legal difficulties, and financial strain were frequently mentioned as relapse triggers, consistent with previous studies that identified external stressors as barriers to sustained recovery [11]. Conversely, patients who remained closely connected to peer groups, therapeutic staff, and/or recovery programs demonstrated greater resilience, supporting the well-documented role of recovery capital in mitigating the risk of relapse [10,12].

Our study further illustrated the dual role of major cultural events in recovery from addiction. On the one hand, the celebratory atmosphere of the 2022 FWC heightened exposure to relapse triggers, particularly in relation to alcohol use, which was the most consistently craved substance. Conversely, the event also provided opportunities for positive social integration. Patients emphasized that the Naufar fan zones and collective match viewing offered safe alternatives to public celebrations, reinforcing abstinence by creating a sense of community belonging and providing meaningful distractions. These findings suggest that large-scale cultural events can serve as both risk factors and recovery catalysts, depending on the eventual presence of structured institutional and social support.

Institutional strategies implemented by Naufar played a pivotal role in shaping these outcomes. Recreational and social programming, including organized group activities and match screenings, contributed to a positive and inclusive atmosphere. Clinical and psychosocial services remained accessible, with participants describing continuous access to therapy, Narcotics Anonymous meetings, and staff availability outside of regular duty hours. These measures align with best practices for comprehensive SUD care, where multidimensional interventions address not only clinical but also social, psychological, and spiritual needs. However, one participant noted reduced medical staffing during the tournament; this observation highlighted the importance of maintaining balance between recreational programming and consistent clinical care during periods of high vulnerability to triggers, cravings and relapse.

### Study Limitations

While this study provides valuable preliminary insights into addictive behaviors during the 2022 FIFA World Cup, some limitations must be acknowledged. Despite achieving thematic saturation, the small, single-center sample (N = 13) limits broader generalizability and strong statistical representation. As with any pilot study, the findings are specific to this cultural and institutional context. Furthermore, the reliance on self-reported data introduces potential social desirability bias regarding clinical services [30]. Future multicenter, longitudinal research with larger cohorts is required to validate these themes and assess long-term impacts.

Lessons learned from our study suggest that treatment centers should not view large cultural events solely as risks to patient stability but rather as opportunities to reinforce recovery and support patients. Proactive institutional strategies, such as establishing safe communal spaces, maintaining flexible therapeutic access, and offering holistic engagement opportunities, can transform potentially triggering contexts into protective ones. At the same time, individualized support remains critical, particularly for patients with longstanding substance use histories or those experiencing persistent psychological distress.

## 5. Conclusions

The experience of patients at the Naufar Center during the 2022 FWC illustrated the complex interplay between environmental factors, institutional strategies, and individual resilience in shaping addiction outcomes during major global sporting events. While major cultural events increase the risk of relapse through heightened exposure to triggers, they can also be an opportunity to strengthen abstinence and social integration when supported by proactive, structured, patient-centered interventions. Our study is the first to report how proactive strategies during major sporting events can support patients with SUDs attending specialized centers for their treatment. Future research is needed to explore how treatment facilities can optimize institutional responses during similar large-scale events, ensuring continuity of care, reinforcing recovery and promoting sustainable abstinence.

## Figures and Tables

**Table 1:** Demographic characteristics of interviewed patients (N = 13).

Characteristic	Category	n	%
Age/years	25–28	4	30.7%
	29–39	6	46.2%
	40 and above	3	23.1%
Marital status	Married	1	7.7%
	Single	9	69.2%
	Divorced	3	23.1%
Education level	Elementary	3	23.1%
	Preparatory	2	15.4%
	Secondary	7	53.8%
	University	1	07.7%
Employment status	Employed	7	53.8%
	Unemployed	6	46.2%
Primary substance Used	Alcohol	2	15.4%
	Methamphetamine	2	15.4%
	Opioids	9	69.2%

**Table 2:** Demographic and professional characteristics of focus group participants (N = 19).

Characteristic	Category	n	%
Gender	Male	17	89.5%
	Female	2	10.5%
Professional role	Physician	7	36.8%
	Clinical psychologist	3	15.8%
	Nursing staff	9	47.4%
Years of clinical experience with patients with SUD	0–5 years	4	21.0%
	6–10 years	9	47.4%
	>10 years	6	31.6%
Primary clinical setting	Inpatient rehabilitation	12	63.2%
	Outpatient clinic	7	36.8%

**Table 3:** Psychological resilience and vulnerabilities.

Feature	Protective Factors (Resilience)	Risk Factors (Vulnerabilities)
Social	Group outings, NA * meetings, and “safe” celebrations.	Old friend groups, environmental availability.
Psychological	Integration through sports, taking the therapist’s advice.	Depression, family stress, and “free time.”
External	Structured Naufar environment.	Financial pressure, legal issues, and overstimulation.

* NA: Narcotics Anonymous.

**Table 4:** Patients’ perception of the Naufar strategy during the 2022 FWC.

Service Type	Strategy Implemented	Patient Impact
Accessibility	24/7 “Open Door” policy and messaging application groups.	Reduced anxiety; P13 felt “accepted with open arms” without an appointment.
Holistic Care	Spiritual trips, gym activities, and volunteering.	Shifted focus from the past to the present; improved mood (P1, P6).
Peer Support	Integration of NA * meetings and recovery coaches.	Created accountability and a “safety in numbers” mentality (P7, P11).

* NA: Narcotics Anonymous.

**Table 5:** Comparative analysis of institutional strategy vs. patient outcome.

Institutional Strategy	Clinical Intent	Patient-Reported Outcome
In-House Fan Zones	Risk mitigation and controlled exposure to festivities.	High engagement; felt “included” without needing tickets or facing triggers (P8, P12).
“Total Environment” Staffing	Constant supervision and immediate craving intervention.	Improved “therapeutic alliance”; patients felt cared for by off-duty staff (P7).
Low-Barrier Access	Proactive outreach (messaging apps/calls) to prevent isolation.	Reduced anxiety; felt “accepted with open arms” even without appointments (P13).
Diversified Programming	Mood stabilization through the gym, spiritual trips, and sports.	Shifted focus from “the past” to present relief and healthy distractions (P1, P6).
Cognitive Reframing	Labeling external temptations as “traps” rather than “pleasure.”	Increased resilience; patients adopted a “sober joy” mindset (P7).

## Data Availability

The data presented in this study are available on reasonable request from the corresponding author.
